# Antimicrobial use practices in canine and feline patients with co-morbidities undergoing dental procedures in primary care practices in the US

**DOI:** 10.1371/journal.pone.0305533

**Published:** 2024-07-10

**Authors:** Maria Soltero-Rivera, Ian Battersby, JoAnn Morrison, Nathaniel Spofford, J. Scott Weese

**Affiliations:** 1 Department of Veterinary Surgical and Radiological Sciences, University of California, Davis, CA, United States of America; 2 Mars Veterinary Health, Vancouver, WA, United States of America; 3 Banfield Pet Hospital, Vancouver, WA, United States of America; 4 Department of Pathobiology, Ontario Veterinary College, University of Guelph, Guelph, ON, Canada; University of Puthisastra, CAMBODIA

## Abstract

This study aimed to investigate how the presence of co-morbid conditions influenced antimicrobial usage as presumptive prophylaxis for suspected bacteremia in dogs and cats undergoing dental treatments at primary care veterinary clinics in the United States. In 2020, data was collected from 1076 veterinary clinics across 44 US states. A total of 681,541 general anesthesia dental procedures were conducted on 592,472 dogs and 89,069 cats. This revealed that systemic antimicrobials were administered in 8.8% of dog procedures and 7.8% of cat procedures in the absence of concurrent periodontal disease or extractions. Cefpodoxime, clindamycin, and amoxicillin-clavulanate were the most frequently used antimicrobials in dogs, while cefovecin, amoxicillin-clavulanate, and clindamycin topped the list for cats. Dogs with cardiovascular, hepato-renal, and endocrine co-morbidities, as well as those undergoing concurrent removal of cutaneous or subcutaneous neoplasia, displayed higher antimicrobial use. Similarly, cats with endocrine or hepato-renal disease, retroviral infection (i.e., feline leukemia virus (FeLV), feline immunodeficiency virus (FIV)), and concurrent removal of cutaneous or subcutaneous neoplasia exhibited increased antimicrobial use. Dogs with hepato-renal abnormalities had longer treatment durations compared to those without (10.1 vs. 9.6 days). Conversely, cats with concurrent removal of cutaneous or subcutaneous neoplasia had shorter durations of treatment as compared to those that did not have this procedure performed (8.4 vs 9.2 days). The findings of this study underscore the necessity for further research and collaboration within the veterinary community to develop evidence-based guidelines, promoting responsible antimicrobial use, and advancing the field of veterinary dentistry for enhanced patient outcomes.

## Introduction

Antimicrobials have become a mainstay in managing oral health conditions and preventing systemic infections in veterinary dentistry. However, with the rising awareness of antimicrobial stewardship and increasing knowledge regarding optimizing the management of patient co-morbidities, the administration of antimicrobials demands careful consideration and tailored approaches. Additionally, concurrent surgical procedures (e. g., removal of cutaneous or subcutaneous neoplasia) during dental procedures pose further challenges in ensuring optimal patient care and antimicrobial stewardship.

Since dental procedures invariably involve trauma to mucosal surfaces, bacterial translocation is presumably relatively common. Study in dogs and cats has been limited but in humans, bacteremia is a common event, occurring in 36–44% of individuals undergoing scaling and root planning, 62–66% undergoing extractions and in 27–28% of dental prophylaxis and probing without scaling and root planning [1, 2]. Bacterial translocation can pose a risk for development of extra-oral infections in some patients, most notably development of infective endocarditis [3]. However, despite the high incidence of bacteremia during routine dental procedures, infectious consequences are rare and are of greatest concern in a small subset of the population with pre-existing and severe cardiovascular abnormalities [2, 4–9]. Accordingly, antimicrobial prophylaxis is not recommended for routine procedures, but is reserved for high risk patients such as those with patent ductus arteriosus, unrepaired cyanotic congenital heart defect, subaortic or aortic stenosis, embedded pacemaker leads or previous infective endocarditis [3]. Veterinary guidance is limited. The American Veterinary Dental College has a more permissive statement that systemic antimicrobials are ‘*recommended to reduce bacteremia for animals that are immunocompromised*, *have underlying systemic disease (such as certain clinically-evident cardiac disease (subaortic stenosis) or severe hepatic or renal disease) and/or when severe oral infection is present*” [10]. The American Animal Hospital Association guidelines indicate that antimicrobials ‘*may be indicated in patients with systemic risk factors*, *such as subaortic stenosis*, *systemic immunosuppression and orthopedic implants placed in the last 12–18 months*” [11]. The British Small Animal Veterinary Association (BSAVA) has updated and released their guidelines for the use of antimicrobials in oral infections. As per the BSAVA antibacterials should be used perioperatively in patients that are immunosuppressed, that have significant comorbidities, when the procedure is long, or there is bony involvement [12].

The objective of this retrospective study was to evaluate antimicrobial use in dogs and cats undergoing routine dental procedures, without periodontal disease or extractions, to understand the influence of underlying health issues such as cardiovascular, endocrine, hepato-renal compromise, as well as feline retroviruses (i.e., feline immunodeficiency virus and feline leukemia virus) on antimicrobial prophylaxis. The focus was on understanding the factors that likely influenced the choice of antimicrobial drugs and treatment durations, in these specific patient groups. We hypothesized that the presence of these specific co-morbid conditions in dogs and cats undergoing professional dental cleanings would be positively correlated with increased antimicrobial usage. Additionally, we expected these co-morbidities would affect duration of antimicrobial treatment. By retrospectively examining real-world scenarios, the study sought to provide valuable information that could lead to more informed and evidence-based guidelines for antimicrobial usage in veterinary dentistry.

## Materials and methods

Information regarding dogs and cats undergoing dental procedures at Banfield Pet Hospital in 2020 was acquired by searching the proprietary medical record system of the practice network, PetWare®. Data on dental procedures, including diagnosis of periodontal disease and the occurrence of extractions, were collected. Animals with a diagnosis of periodontal disease or those that underwent extractions were intentionally excluded from the dataset to exclude their potential influence on prescribing practices.

Co-morbidity data, structured as clinical diagnostic codes, were obtained, and categorized into cardiovascular, endocrine, hepato-renal compromise, retroviral status (cats only), history of previous tibial plateau levelling osteotomy (TPLO) (dogs only) and whether concurrent cutaneous or subcutaneous neoplastic mass removal was performed at the time of the dental cleaning (Table 1). Clinical diagnoses were limited to those which would be more readily identified and attained, and more commonly seen in primary care practice. Structured pharmaceutical data included drug name, concentration, route of administration, dose, and duration of treatment. Animal species, age, and weight were also collected. Written consent for medical data analysis was obtained from clients for every pet included in the analysis, prior to treatment. Institutional Review Board approval was not required for this study as there is no access to client data and this study qualifies as quality assurance and quality improvement activities.

**Table 1:** Reasons for anti-biotherapy evaluated and frequency of code recalls per code searched in 2020. Reasons for anti-biotherapy (i.e., co-morbidities) were grouped into six major categories as shown here. For the cardiovascular group, diagnoses more likely associated with a cardiac murmur were included. In the endocrine group, diagnoses historically associated with immune dysfunction were included. The hepato-renal compromise category was limited to hepatic and renal diseases due to the ability to identify bloodwork abnormalities and the prevalence in the population. Feline retroviral diseases (feline leukemia (FeLV) and immunodeficiency virus (FIV)) considered were those associated with immune dysfunction. Tibial plateau leveling osteotomy (TPLO) was included as this procedure involves placement of a long-term surgical implant. Cutaneous or subcutaneous neoplastic mass removal procedures were included as this was a common concurrent surgical procedure performed with dental prophylaxis.

Category	Diagnosis/Structured line item	Total number
**Cardiovascular**	• Murmur	177,828
	• Murmur, acquired	3,035
	• Murmur, congenital	864
	• Murmur, physiologic	436
	• Endocarditis	25
	• Mitral insufficiency	2,433
	• Tricuspid insufficiency	476
	• Aortic stenosis	286
	• Pulmonic stenosis	222
	• Hypertrophic cardiomyopathy	502
	• Restrictive cardiomyopathy	17
**Endocrine**	• Hyperadrenocorticism/Cushing’s	7,619
	• Cushing’s Disease, Iatrogenic	434
	• Diabetes Mellitus	16,969
**Hepato-renal compromise**	• Renal Disease, Chronic	32,582
	• Pyelonephritis	296
	• Azotemia	5,260
	• Azotemia, Renal	4,110
	• Uremia	236
	• Iris Stage 1	286
	• Iris Stage 2	923
	• Iris Stage 3	411
	• Iris Stage 4	156
	• Hepatopathy	97,791
	• Hepatitis, Chronic Active	75
	• Hepatitis	829
	• Portosystemic shunt	537
	• Hepatic Lipidosis	383
	• Hepatic Failure, Chronic	286
	• Hepatic Disease, Conservative	916
	• Hepatic Disease, Extensive	452
	• Biliary Mucocele	196
	• Cholecystitis	214
	• Cholelithiasis	286
	• Cholangitis	401
	• Cholangiohepatitis	N/A
**Feline retroviral**	• Feline leukemia virus	1633
	• Immunodeficiency virus, feline	4,584
**Presence of orthopedic implants**	• TPLO inventory line items	70
**Cutaneous or subcutaneous neoplasia**	• Mass removal inventory line items	35,131

Univariable analysis was performed using chi squared analysis for categorical data and logistic regression for continuous data. Multivariable analysis was performed using stepwise forward logistic regression. Variables with a P<0.20 were entered into the model, with the final threshold for significance set at P≤0.05. Insignificant variables were not retained in the model unless they were deemed to be confounders. Confounders were identified by observing the changes in coefficients in other variables after removing the target variable. The confounder was forced into the final model if a change of >10% occurred for any variable. Two-way interactions were tested and were retained in the model if they were deemed significant. Odds ratios and 96% confidence intervals were calculated. Model fit was evaluated using the Whole Model test. Standard least squares analysis was used to evaluate factors associated with treatment duration. Analysis was performed using JMP (SAS Institute, Cary, NC, USA).

## Results

Data were obtained for 681,541 dental cleanings (no extractions and no periodontal disease noted), performed on 592,472 dogs and 89,069 cats at 1076 veterinary clinics in 44 US states. Systemic antimicrobials were administered in 51,986 (8.8%) procedures in dogs and 6936 (7.8%) in cats. The most common antimicrobials and antimicrobial combinations used are presented in Table 2.

**Table 2:** Most common antimicrobials administered to dogs (n = 51,986) and cats (n = 6,936) that underwent dental procedures. These patients were not diagnosed with periodontal disease, and no extractions were performed during their procedures. Other less commonly used antimicrobials were prescribed to 769 dogs and 127 cats.

Dogs		Cats	
Antimicrobial	n (%)	Antimicrobial	n (%)
Cefpodoxime	14,274 (27%)	Cefovecin	3654 (52%)
Clindamycin	13,176 (25%)	Amoxicillin-clavulanate	1562 (22%)
Amoxicillin-clavulanate	9,913 (19%)	Clindamycin	1067 (15%)
Cefovecin	4,884 (9.2%)	Amoxicillin	245 (3.5%)
Amoxicillin	3,176 (6.0%)	Marbofloxacin	83 (1.2%)
Metronidazole	2,720 (5.1%)	Metronidazole	67 (1.0%)
Doxycycline gel	1,337 (2.5%)	Doxycycline gel	50 (0.7%)
Marbofloxacin	763 (1.4%)	Cefazolin	29 (0.4%)
Doxycycline	655 (1.2%)	Doxycycline	26 (0.4%)
Cefazolin	319 (0.6%)	Cefpodoxime	26 (0.4%)

Overall, 82,064 (92%) of cats had none of those co-morbidities described in Table 1 while 6,545 (7.3%) had one, 449 (0.5%) had two and 11 (0.012%) had three. In dogs, 520,948 (88%) had none of those co-morbidities described in Table 1 while 66,400 (11%) had one, 4,811 (0.8%) had two, 304 (0.05%) had three and nine (0.002%) had four. Not all cats and dogs with comorbidities were treated with antimicrobials.

**Fig 1:**
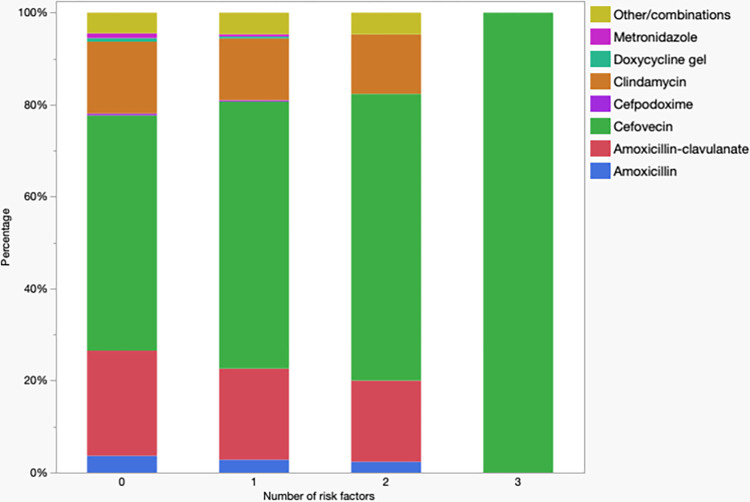
Antimicrobial used in cats (n = 6,936) underdoing dental prophylaxis by number of co-morbidities. Organ refers to patients with hepato-renal dysfunction.

**Fig 2:**
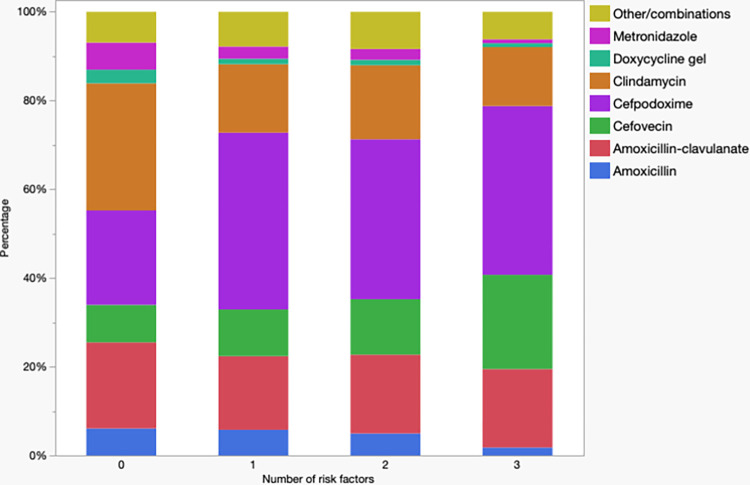
Antimicrobial used in dogs (n = 51,986) underdoing dental prophylaxis by number of co-morbidities. Organ refers to patients with hepato-renal dysfunction. Dogs with four risk factors were excluded because of the small sample size.

There were different patterns of antimicrobial use when evaluated based on the combined number of different co-morbidities (i.e., hepato-renal, cardiovascular, endocrine, retroviral infection, TPLO, cutaneous or subcutaneous neoplasia removal). Antimicrobial use by number of co-morbidities (excluding dogs with four because of the small sample size) is presented in Fig 1 (cats) and Fig 2 (dogs). In cats, a marked difference in antibiotic choice was noted in patients with three co-morbidities with all patients receiving cefovecin. In dogs, on the other hand, a difference can be noted between patients with none to one co-morbidity with noticeable increase in use of cefpodoxime. Cefovecin use was also increased in dogs with three co-morbidities however, different from cats, this wasn’t the only antimicrobial used.

### Univariate analyses of association with systemic antimicrobial administration

The univariable analyses presented in Tables 3 and 4 notes significant associations between antimicrobial administration and reported co-morbidities in both cats and dogs, respectively.

**Table 3:** Univariable analysis of associations with systemic antimicrobial administration in cats that underwent dental procedures (n = 89,069). Cats were without a diagnosis of periodontal disease and extractions were not performed.

Co-morbidities	Systemic antimicrobials (n/N, %) administered to animals with the comorbidity)	Systemic antimicrobials (n/N, %) administered to animals without the comorbidity)	*P* value
Cardiovascular	233/2,225 (10.5%)	6,703/80,141 (7.7%)	<0.0001
Hepato-renal compromise	278/2,437 (11.4%)	6,658/86,632 (7.7%)	<0.0001
Endocrine	84/597 (14.1%)	6,852/88,472 (7.7%)	<0.0001
Feline Retroviral infection	137/971 (14%)	6,799/88,098 (7.7%)	<0.0001
Cutaneous or subcutaneous neoplasia removal	441/1,246 (35.4%)	6,495/87,823 (7.4%)	<0.0001

**Table 4:** Univariable analysis of associations with systemic antimicrobial administration in dogs that underwent dental procedures (n = 592,472). Dogs were without a diagnosis of periodontal disease and extractions were not performed.

Co-morbidities	Systemic antimicrobials (n/N, %) administered to animals with the comorbidity)	Systemic antimicrobials (n/N, %) administered to animals without the comorbidity)	*P* value
Cardiovascular	2,668/19,281 (14%)	49,318/573,191 (8.6%)	<0.0001
Hepato-renal compromise	2,420/20,782 (12%)	49,566/571,690 (8.8%)	<0.0001
Endocrine	395/2,706 (15%)	51,591/589,766 (8.7%)	<0.0001
TPLO surgery	13/70 (19%)	51,973/592,402 (8.8%)	0.004
Cutaneous or subcutaneous neoplasia removal	11,883/34,131 (35%)	40,103/558,341 (7.2%)	<0.0001

## Multivariate analyses of association with systemic antimicrobial administration

### Impact of age and weight on antimicrobial administration

Elderly and underweight cats were administered antimicrobials, with an average age of 8.4 years (Standard Deviation (SD) 4.1) and weight of 5.2 kg (SD 1.4), respectively. In contrast, cats without antimicrobials were younger, with an average age of 7.7 years (SD 3.8), and slightly heavier, with an average weight of 5.4 kg (SD 1.4) (both P<0.0001). Endocrine or hepato-renal compromise, retroviral infection and concurrent cutaneous or subcutaneous neoplasia removal (i.e., performed at the time of dental prophylaxis) were associated with antimicrobial use in the multivariable model (Table 5).

**Table 5:** Multivariable analysis of associations with systemic antimicrobial administration in dogs (n = 592,472) and cats (n = 89,069) undergoing dental procedures in the absence of a diagnosis of periodontal disease and extractions. Weight and age were evaluated as continuous data and presence of co-morbidities was evaluated as categorical data. Only statistically significant variables are shown.

Species	Variable	Odds ratio (95% CI)	*P* value
Dogs	Weight (kg)	0.999 (0.999–1.000)	0.0004
	Age (years)	1.08 (1.08–1.09)	<0.0001
	Cardiovascular	1.29 (1.23–1.35)	<0.0001
	Endocrine	1.22 (1.09–1.37)	0.0006
	Hepato-renal compromise (i.e., renal, hepatic)	1.05 (1.00–1.10)	0.034
	Cutaneous or subcutaneous mass removal	6.2 (6.03–6.34)	<0.0001
Cats	Weight (kg)	0.93 (0.91–0.94)	<0.0001
	Age (years)	1.04 (1.03–1.05)	<0.0001
	Endocrine	1.78 (1.41–2.26)	<0.0001
	Hepato-renal compromise (i.e., renal, hepatic)	1.19 (1.05–1.36)	0.009
	Cutaneous or subcutaneous mass removal	6.3 (5.61–7.14)	<0.0001
	Retroviral infection	1.96 (1.63–2.36)	<0.0001

Older dogs and dogs that weighed more received antimicrobials with a mean of 8.4 years of age (standard deviation (SD) 3.3 y) and 16.6 kg (SD 13.1 kg) while those that did not were a mean of 7.2 years (SD 3.2 y) and 16.3 kg (SD 12.9) (both *P*<0.0001). Cardiovascular, hepato-renal compromise and endocrine comorbidities, as well as concurrent cutaneous or subcutaneous mass removal were associated with antimicrobial use in dogs in the multivariable analysis (Table 5).

### Impact of variables on duration of treatment

Only cutaneous or subcutaneous mass removal was associated with treatment duration in cats (*P* = 0.008). Counterintuitively, duration of treatment was shorter in cats that underwent concurrent cutaneous or subcutaneous mass removal as compared to those who did not have a cutaneous or subcutaneous mass removed at the time of the dental procedure (mean 8.4 vs 9.2 days).

Cardiovascular and hepato-renal compromise comorbidities, as well as concurrent TPLO, were associated with variations in treatment duration in dogs (all *P*<0.0001). Compared to patients without co-morbidities, duration was longer in dogs with hepato-renal abnormalities (10.1 vs 9.6 days) but shorter in dogs with cardiovascular comorbidities (9.2 vs 9.6 days) and in dogs that had previously undergone TPLO (8.1 vs 9.6 days).

### Antimicrobial administration for different co-morbidities

**Fig 3:**
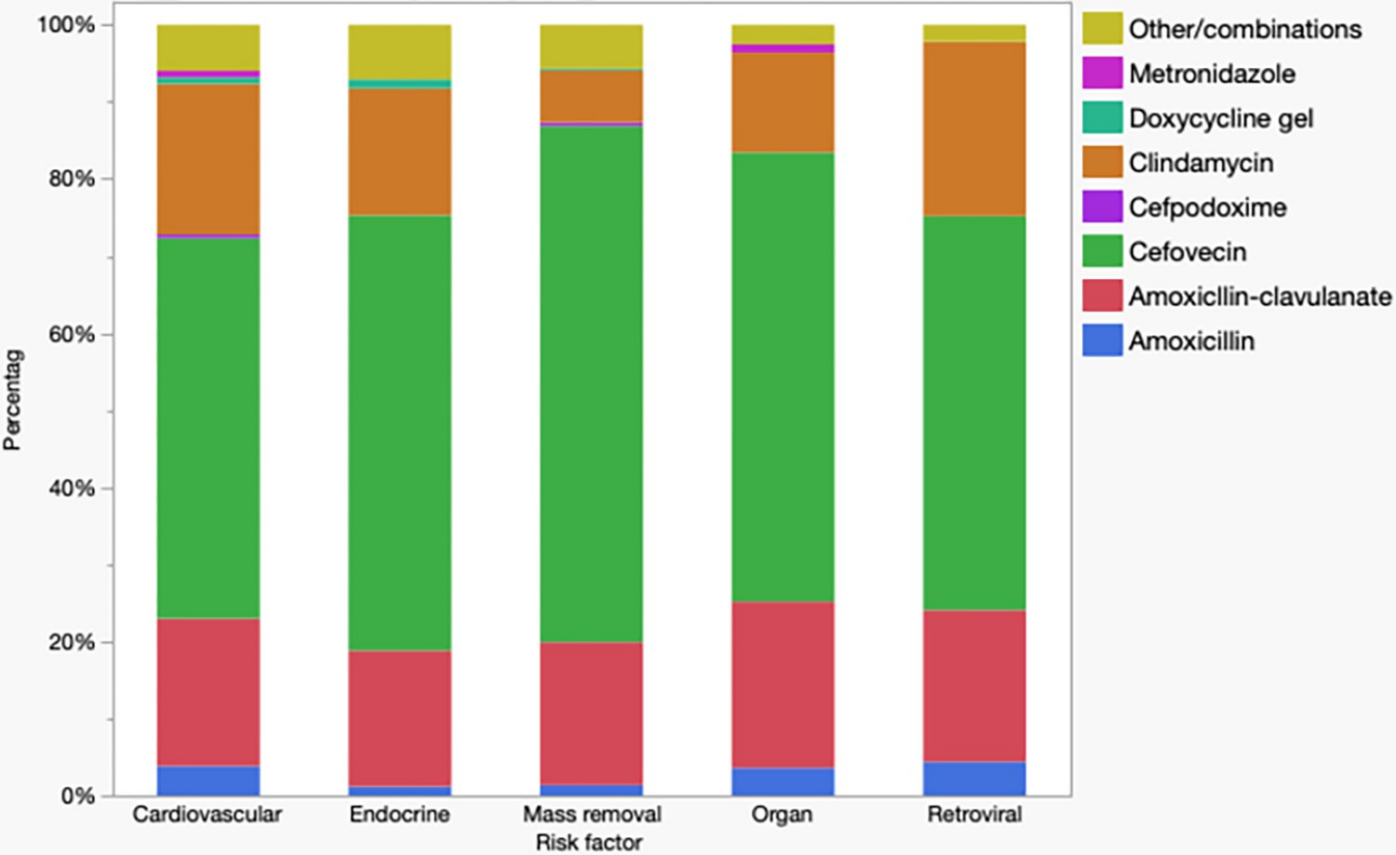
Relative use of antimicrobials for cats underdoing dental prophylaxis (n = 6,936) with different co-morbidities.

**Fig 4:**
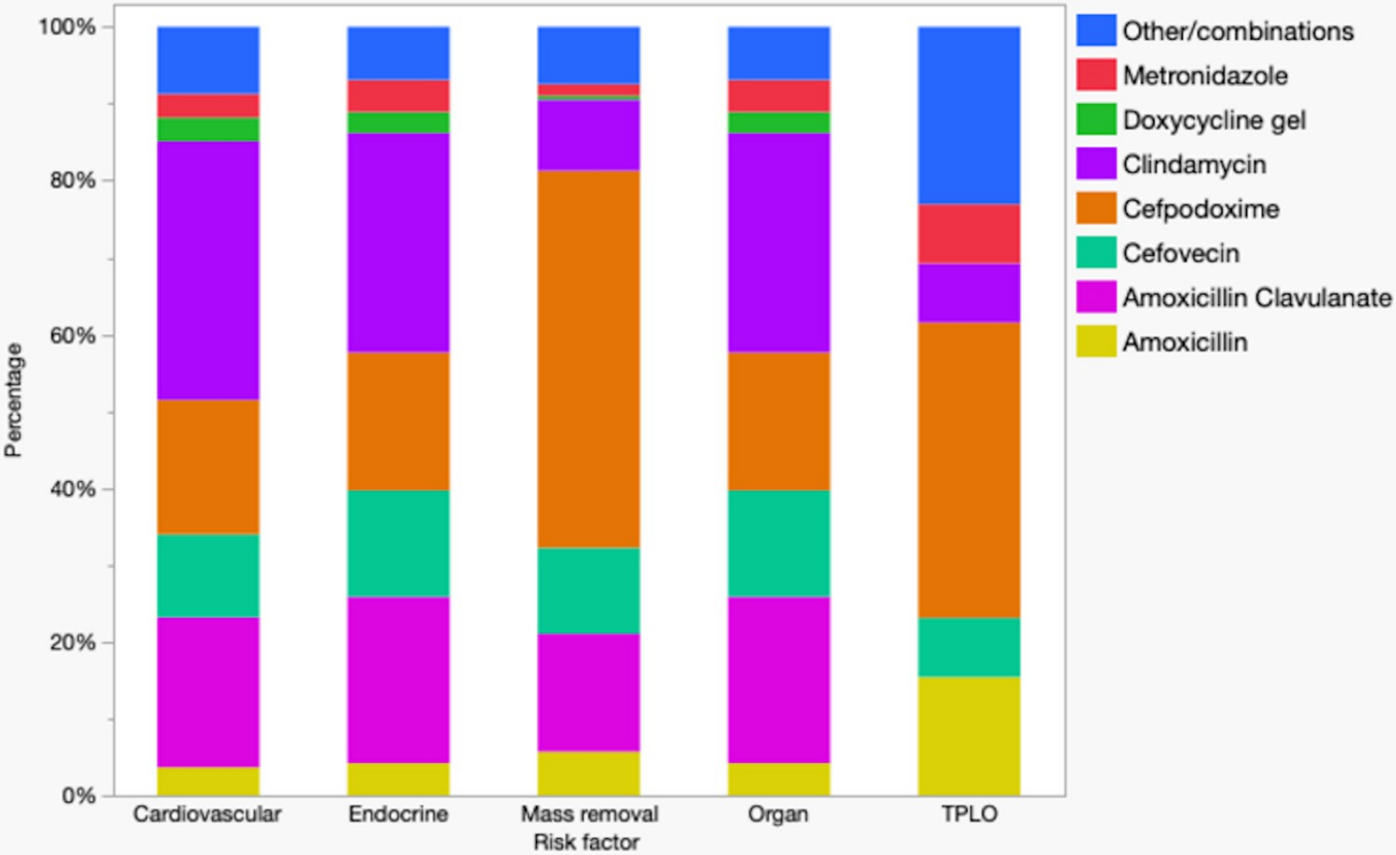
Relative use of antimicrobials for dogs undergoing dental prophylaxis (n = 51,986) with different for co-morbidities.

Relative use of antimicrobials for different co-morbidities for cats and dogs is depicted in Figs 3 and 4, respectively. Fig 3 shows all cats with co-morbidities were mainly treated with cefovecin. Fig 4 shows that in dogs with cardiovascular, endocrine, and hepato-renal compromise treatment with clindamycin predominated whereas in those undergoing cutaneous or subcutaneous mass removal of with a history of TPLOs treatment with cefpodoxime predominated.

In the analysis focusing on dogs and cats treated with antimicrobials, several statistically significant associations were observed between specific antimicrobials and clinical factors (Table 6). In cats, cutaneous or subcutaneous mass removal at the time of dental prophylaxis was positively associated with the use of cefovecin and negatively associated with the use of clindamycin and amoxicillin. On the other hand, retroviral infection was positively associated with the use of clindamycin. In dogs, cutaneous or subcutaneous mass removal at the time of dental prophylaxis was positively associated with the use of cefovecin and cefpodoxime, while use of clindamycin, amoxicillin, amoxicillin clavulanate and metronidazole were negatively associated. In dogs with hepato-renal compromise comorbidities, the use of amoxicillin, amoxicillin-clavulanate and metronidazole were positively associated whereas clindamycin and cefpodoxime were negatively associated. Additionally, cardiovascular diseases in dogs were positively associated with use of clindamycin but negatively associated with amoxicillin and cefpodoxime administration (Table 6).

**Table 6:** Positive and negative associations with antimicrobial administration in dogs and cats. Only statistically significant results are provided (* = P<0.0001).

	Cutaneous or subcutaneous mass removal		Cardiovascular	Hepato-renal compromise (i.e., renal, hepatic)	Retroviral disease (FeLV, FIV)
Antimicrobial	Dog	Cat	Dog only	Dog only	Cat only
Cefovecin	OR 1.7, 95% CI 1.6–1.8*	OR 2.0, 95% CI 1.6–2.5*			
Clindamycin	OR 0.27, 95R CI 0.26–0.29*	OR 0.40, 95% CI 0.28–0.58*	OR 1.13, 95% CI 1.04–1.24, (P = 0.004)	OR 0.77, 95% CI 0.70–0.85*	OR 1.56, 95% CI 1.04–2.35 (P = 0.031)
Amoxicillin	OR 0.79, 95% CI 0.72–0.86*	OR 0.39, 95% CI 0.17–0.87 (P = 0.022)	OR 0.80, 95% CI 0.65–0.98 (P = 0.031)	OR 1.6, 95% CI 1.34–1.86*	
Amoxicillin Clavulanate	OR 0.90, 95% CI 0.85–0.95 (P = 0.0003)			OR 1.2, 95% CI 1.04–1.3 (P = 0.005)	
Cefpodoxime	OR 3.6, 95% CI 3.5–3.8*		OR 0.86, 95% 0.77–0.95 (P = 0.005)	OR 0.79, 95% CI 0.71–0.88*	
Metronidazole	OR 0.30, 95% CI 0.26–0.34*			OR 2.2, 95% CI 1.9–2.5*	

### Intravenous antimicrobial administration

Peri-operative intravenous antimicrobials were uncommonly used, being only administered to 1040/53,323 (2.0%) of dogs and 44/6986 (0.6%) of cats that received antimicrobials. When the analysis was performed, including only animals that received antimicrobials, in dogs, cardiovascular (OR 1.6, 95% CI 1.25–1.99, P<0.0001) or hepato-renal (OR 1.34, 95% CI 1.04–1.73, P = 0.023) comorbidities, as well as cutaneous or subcutaneous mass removal (OR 2.05, 95% CI 1.8–2.3, P<0.0001), were found to be associated with the use of intravenous antimicrobials. In cats, hepato-renal comorbidity was the only variable associated with intravenous antimicrobial use compared other routes of administration (OR 2.9, 95% CI 1.1–7.5, *P* = 0.027).

## Discussion

The discussion on antimicrobial use in veterinary dentistry, considering patient co-morbidities, highlights intriguing associations. A noteworthy aspect of the study is the association of different drugs with specific co-morbidities and risk factors. The study also reveals longer treatment durations in dogs with hepato-renal compromise than those without. In all these cases, clinicians may exhibit concern regarding the elevated risk of bacterial infections arising from the introduction of bacteria into the bloodstream during dental procedures or from pre-existing bacterial colonization in the oral cavity. However, the true risk of clinical bacterial infections from dental procedures remains unknown in veterinary practice.

In the realm of veterinary dentistry, an important aspect to consider is the absence of established guidelines concerning antibiotic usage during dental procedures for patients with co-morbidities in the USA [13, 14]. Understandably, veterinarians may err on the side of caution out of a well-founded desire to prioritize patient safety and mitigate risks. Since antimicrobials are not innocuous, direct evidence of a benefit is lacking and comparative data from humans does not support such widespread use of antimicrobials, a ‘least harms’ approach could be considered instead [3, 5, 7, 8, 15–19].

Multiple comorbidities were associated with an increased likelihood of antimicrobial use in our cohorts. It is important to consider that not every animal with indications of possible co-morbidities will undergo full evaluation to obtain a definitive diagnosis prior to dental treatment under anesthesia. Consequently, at times the risk is not completely understood prior to treatment. However, despite that, most animals with comorbidities did not receive antimicrobial prophylaxis, an encouraging finding given the presumed lack of need for antimicrobials in most situations [2, 4–9]. While there are contrasts between human and veterinary dentistry, the pathophysiology of infection concerns is likely very similar. Therefore, it is reasonable to consider human guidelines. These tend to restrict antimicrobial use to a very narrow subset of the population and use short treatment durations [3, 5, 7, 15–19].

Many dogs received antimicrobials in the presence of a clinical diagnosis of a cardiovascular disease. The 2007 American Heart Association guidelines, endorsed by the American Dental Association, Infectious Diseases Society of America and Pediatric Infectious Diseases Society, recommend prophylaxis of a relatively small population of patients, namely those with a prosthetic heart valve, previous infective endocarditis, unrepaired cyanotic congenital heart disease, completely repaired heart defect with prosthetic material placed within the past 6 months, repaired congenital heart diseases with residual defects at the site or heart transplant recipients with valvulopathy [3]. These conditions would apply to very few veterinary patients.

The impetus to treat cats with antimicrobials was seen in association with retroviral infections. Retroviral infections such as feline leukemia virus and immunodeficiency virus can compromise the immune system making cats more susceptible to opportunistic bacterial infections, such as oral, respiratory, and urinary tract infections. However, the degree of immunocompromise can vary, prompting questions about the perceived immunocompromised state and whether it is warranted in most cases [20, 21]. In humans with human immunodeficiency virus (HIV), routine peri-dental administration of antimicrobials are not recommended unless extractions are performed in patients with neutropenia (i.e., absolute neutrophil counts that are <500 cells/mm3) [15, 22].

Pre-existing endocrine disease was also associated with increased likelihood of antimicrobial administration in both dogs and cats. Routine antimicrobial prophylaxis is not recommended in humans with endocrinopathies [16]. When evaluating the prevalence of bacteriuria in dogs with hypercortisolism, this is lower than expected with only 18% tested positive and of those 83% were subclinical [23]. A single case report detailing local and systemic complications following dental extractions in a cat with diabetes exists; however, this patient was not well controlled and risk cannot be ascertained from a single case [24]. Taken together, the level of immunocompromise caused by these endocrine diseases, if well-controlled, is likely negligible and there may be a risk of over usage of antimicrobials in these patients. The level of clinical control over the endocrine disease was not evaluated in this study, so it is uncertain if patients were well controlled or poorly controlled at the time of the dental cleaning.

Hepato-renal compromise at the time of dental procedures were also noted to be risk factors of the administration of antimicrobial prophylaxis in both dogs and cats. Prophylactic antibiotics are recommended prior to invasive dental treatment in human patients with nephritic syndrome and kidney transplants; however, in chronic kidney disease recommendations for this vary [17]. Antimicrobial prophylaxis is also suggested in patients with end stage liver dysfunction in patients with ascites and spontaneous bacterial peritonitis [18]. However, a large retrospective study of patients with liver transplants undergoing extractions concluded that there is no need for antimicrobial prophylaxis in that patient group as long as the technique is atraumatic [19]. These scenarios in which antimicrobial prophylaxis is used in human medicine are not commonly seen in primary care veterinary practice and consequently, this may represent an area where significant reductions in antimicrobial use could be targeted, without negatively impacting patient care.

Concurrent cutaneous or subcutaneous mass removal was a relatively common event. This is an area where there is no ability to extrapolate from human dentistry, as those procedures would not be linked in humans. Presumably, the reason that antimicrobials were more likely to be used in animals undergoing concurrent dental procedures and cutaneous or subcutaneous mass removal was concern about bacteremia and aerosolization of bacteria from the dental procedure resulting in infection at the surgical site [2]. No data are available regarding the incidence of infection when combining these types of procedures. However, general surgical principles support avoiding combining clean with contaminated procedures considering that the mouth is populated by many bacteria in health and disease that may be harmful for the skin when undergoing an insult (i.e., surgery). This area requires further study due to its common occurrence, likely attributed to convenience, concern regarding non-compliance, and the desire to avoid multiple anesthetic procedures.

Prevalence data regarding bacteremia in dogs and cats undergoing routine dental procedures are limited but the bacteria involved have been described. Blood cultures from patients undergoing these procedures have revealed various species, mostly a diverse range of oral commensals with varying disease risks. Among dogs undergoing dental procedures, the incidence of anaerobic bacteremia was higher (43%) than that of gram-positive aerobic (29%), and gram-negative aerobic (29%) bacteremia [2, 25–27]. However, bacteremia has also been documented to occur when tooth brushing and while chewing and in a healthy human patients with a working reticuloendothelial system this is inconsequential [28, 29]. Despite the high prevalence of bacteremia, clinical consequences appear to be limited in the general patient population [30]. Further, while 12% of dogs and 8% of cats had one or more co-morbidities of interest and those were associated with an increased likelihood of antimicrobial administration, rarely would antimicrobial prophylaxis be recommended for those comorbidities in human guidelines [31]. While veterinary data are lacking, there is no published evidence to support that dogs and cats would be at increased risk of disease following transient bacteremia compared to human counterparts.

In most cases, the lack of evidence supporting the need for antibiotics raises a critical discussion point. However, there also needs to be consideration for appropriate coverage if antibiotic use is indicated. Increased clindamycin use for cardiovascular conditions would offer appropriate coverage due to concerns of streptococcal and staphylococcal infections, even though it sacrifices gram-negative coverage. However, this would be indicated only in severe cardiovascular disease which is of rare occurrence in veterinary medicine [3, 32]. Similarly, the use of metronidazole to prevent secondary anaerobic infections [32] in distant organs would provide adequate coverage; however, as discussed above cases that would actually benefit from this are rarely seen in primary care practice. On the other hand, the use of third generation cephalosporins (such as cefpodoxime) for cutaneous or subcutaneous mass removal may be deemed excessive [4, 32], as is the use of a higher tier broad spectrum antimicrobial in patients without evidence of periodontal disease or those requiring tooth extractions.

In humans, penicillins are the main drugs recommended for prophylaxis, with other options such as macrolide and aminoglycosides used in some situations [7, 8]. A variety of antimicrobials were identified in this study. Amoxicillin use would be consistent with typical human guidelines, but it accounted for <10% of use here [28]. Cefovecin was commonly used, which raises concerns because of its higher tier status and long duration of action, as well as limited activity against *E*. *coli* [33]. Cefpodoxime was also commonly used, with similar concerns about the necessity of this higher tier drug [34]. Cefovecin and cefpodoxime are administered every two weeks or once a day, respectively, thus administration factors could also play a role in this selection. Amoxicillin-clavulanate is lower tier option with excellent activity against common pathogens. Clindamycin is often used for oral disease because of its activity against staphylococci and streptococci, and anaerobes [35]. Additionally, the surprisingly low use of amoxicillin raising questions about its potential underutilization in veterinary dentistry, particularly in situations such as a tibial plateau leveling osteotomy (TPLO), where it would typically be an ideal antimicrobial selection due to the risk of staphylococcal infections [36].

Various impacts of different comorbidities on treatment duration were identified in both dogs and cats. However, analysis of large datasets can identify significant but clinically irrelevant differences. Here, the differences in duration were likely negligible from clinical and stewardship standpoints. However, they might provide some insight into clinicians’ decision-making processes. One important area that could not be specifically evaluated here was timing of administration. Prophylaxis should be administered such that therapeutic drug levels are present during the period of risk. Accordingly, antimicrobials should be administered shortly before the procedure, ideally intravenously 30–60 minute before the procedure, and typically do not need to be continued after [4]. Administration of antimicrobials after the procedure could be minimally effective as the main period of risk would have passed. The uncommon use of intravenous antimicrobials, that would more likely have been administered prior to the procedure, indicates a need to better understand how antimicrobials are used, when they are used. Both unnecessary use and suboptimal use are concerns from patient care and antimicrobial stewardship standpoints. Development and use of clinical guidelines could improve both of those areas.

Overall, this study offers valuable insights into the thought processes behind drug selection based on patient co-morbidities and risk factors in veterinary dentistry. The findings suggest opportunities for improvement and education to refine antimicrobial approaches, ensuring optimal care for patients undergoing dental procedures with varying health conditions. Further research and discussion within the veterinary community are necessary to develop evidence-based guidelines and promote the responsible use of antimicrobials in dental care, ultimately advancing the field of veterinary dentistry and patient outcomes.
